# Promoting Flexibility in Expectations: A Randomized-Controlled Online-Intervention Study for Mild Psychopathological Symptoms

**DOI:** 10.32872/cpe.15079

**Published:** 2026-05-29

**Authors:** Anne-Catherine I. Ewen, Marcel Wilhelm

**Affiliations:** 1 Department of Clinical Psychology and Psychotherapy, Philipps-University of Marburg, Marburg, Germany; RPTU University Kaiserslautern-Landau, Landau, Germany

**Keywords:** expectation violation, immunization, ViolEx-model, expectation focused psychological intervention EFPI, online interventions, subclinical to mild symptoms

## Abstract

**Background:**

Research demonstrates that mental disorders are associated with specific dysfunctional expectations and a reduced ability to adjust them, even after expectation-violating experiences. The *ViolEx* (violated expectation) model offers a framework to explain why expectations persist or change, introducing the concept of *cognitive immunization* as a potential explanation for differences in information processes. Expectation-focused psychological interventions (EFPI) aim to promote expectation adaptation.

**Method:**

This study examines the effectiveness of an online EFPI platform for individuals with mild depressive and/or anxiety symptoms. A total of 128 participants, screened with the PHQ-9 (scores 5-9) and/or BAI (scores 8-25), were randomly assigned to one of three groups. The EFPI group and the active control group (ACG) received a psychoeducational video about expectations and their influence on behavior. Over four weeks, the EFPI group completed behavioral experiments to test their personal expectations, while the ACG used only cognitive strategies to challenge personal expectations. A third group (control group; CG) received no intervention. Surveys were administered at baseline, four weeks, and eight weeks after the initial assessment.

**Results:**

A significant reduction in cognitive immunization was observed over measurement timepoints, with a significant difference to the CG at the follow-up. Anxiety symptoms appear to moderate this effect, whereas EFPI did not influence depressive symptoms, nor did depressive symptoms moderate changes in cognitive immunization.

**Conclusion:**

This study is the first to evaluate online EFPI for mild depression and/or anxiety symptoms, suggesting that EFPI may reduce cognitive immunization. Future studies should investigate therapist-delivered EFPI in clinical populations with more severe symptoms.

## Highlights

- Dysfunctional expectations and cognitive immunization maintain mental disorders by blocking belief/expectation updating.
- Online expectation-focused psychological interventions (EFPI) can reduce cognitive immunization over time.
- EFPI effects appear stronger in individuals with elevated anxiety symptoms.
- EFPI is promising, but more therapist-guided and clinical research is needed.

Research has increasingly focused on non-specific factors in psychotherapy, known as *common factors* (Wampold & Imel, 2015). These include concepts such as the therapeutic relationship, activation of resources, actualization of the patient's problems, motivational clarification, and problem solving, as well as alliance, empathy, expectations, cultural adaptation, and therapist differences (Wampold, 2015; Wampold & Imel, 2015). One promising factor receiving growing attention is the role of expectations in the therapeutic process (Constantino et al., 2011, 2012; Greenberg et al., 2006). It is well-established that expectations significantly influence therapy outcomes. Placebo research highlights the power of treatment expectations (Bingel, 2020; Evers et al., 2018; Kirsch, 2018; Kirsch et al., 2016; Wampold et al., 2005), and specific expectations have been shown to impact therapeutic relationships (Al-Darmaki & Kivlighan, 1993; Finsrud et al., 2022; Wright & Davis, 1994), proper self-efficacy (Bandura, 1977; Lightsey, 1999), or the effectiveness of different intervention techniques (Craske et al., 1988; Rief & Glombiewski, 2017).

In clinical psychology research, studies suggest that individuals with mental disorders not only hold more dysfunctional expectations but also struggle to adjust their expectations after disconfirming experiences (Kirchner et al., 2022; Kube, Kirchner, et al., 2019; Kube et al., 2020; Rief & Joormann, 2019). In this context, dysfunctional expectations are defined as dysfunctional thoughts that are unhelpful, distorted, idiosyncratic, and negatively biased (Lam & Cheng, 2001). Not only should the content of a certain expectation be considered, but also information processing factors should be explicitly addressed that are responsible for expectation origination, maintenance, and modification.

The concept of expectation is also prominent in action planning and decision-making models (Atkinson & Feather, 1966; Kahneman & Tversky, 2013). A similar generalized model called the ViolEx (violated expectation) model was developed, offering a framework for understanding rigid expectations as core features of mental disorders (Rief et al., 2015). According to this model, generalized expectations are shaped by past experiences, social influences, and individual differences (Gollwitzer et al., 2018; Rief et al., 2015). These generalized expectations lead to situation-specific predictions, which may be confirmed or violated by experience. Healthy individuals generally adjust their expectations following disconfirming experiences. However, research suggests that in mental disorders such as depression, expectation updating may occur less frequently or require stronger disconfirmatory evidence to take place. A key mechanism for maintaining dysfunctional expectations, as proposed by the ViolEx model, is *cognitive immunization* (Gollwitzer et al., 2018; Kube, Rief, et al., 2019; Pinquart et al., 2021). This involves a reappraisal of an expectation-violating experience to preserve the original expectation, often by dismissing disconfirming events as exceptions (“*it was only an exception”).* A well-known process in cognitive-behavioral theories that contributes to the persistence of dysfunctional expectations or beliefs is cognitive or behavioral avoidance (Aldao et al., 2010; Hofmann & Hay, 2018; Servatius, 2016).

In the treatment of anxiety disorders, dysfunctional expectations such as *“Something bad will happen”* or “*They will laugh at me”* are addressed explicitly through exposure therapy, which aims to reduce avoidance behavior and promote expectation adjustment (Clark, 1999; Craske et al., 1988; Craske et al., 2014). Research indicated that individuals with mental disorders exhibit not only increased dysfunctional expectations but also difficulty in accommodating these dysfunctional expectations after new expectation-disconfirming experiences (Kube, D’Astolfo, et al., 2017; Rief & Joormann, 2019). In depression, for instance, patients often struggle to change negative performance expectations even after experiencing positive outcomes (Kube, Rief, et al., 2019; Kube, Rief, & Glombiewski, 2017).

These findings suggest that persistent dysfunctional expectations and impaired adaptation processes may be central to various mental disorders. Addressing these mechanisms directly in therapy could foster therapeutic change (Craske et al., 2014). Expectation focused psychological interventions (EFPI) in psychotherapy has been proposed as a strategy to address dysfunctional expectations by fostering conscious awareness, encouraging behavioral testing of expectations, and promoting expectation-disconfirming experiences (Kube, Glombiewski, & Rief, 2019; Rief & Glombiewski, 2016).

This study investigates the effectiveness of online EFPI in reducing cognitive immunization processes in individuals with mild depressive and/ or anxious symptoms. The intervention aims to encourage participants to consciously explore how expectations influence their feelings and behaviors, as well as actively test these expectations to foster adaptive changes.

In this online randomized controlled trial, mildly depressed or anxious participants were assigned to one of three groups. The experimental group (EFPI group) received a psychoeducational video and conducted behavioral experiments to test personally burdensome expectations over four weeks. By 'burdensome expectations,' we refer to expectations that cause psychological distress or hinder goal achievement.

The active control group (ACG) received the same psychoeducational video but was only asked to observe personal expectations over four weeks. The control group (CG) received no interventions. It was assumed that the EFPI group would experience a significant reduction in cognitive immunization over time, with the greatest reduction compared to the ACG and CG. Additionally, it was expected that symptom severity, e.g., depressive and anxiety symptom severity, would moderate the effect of the online interventions on cognitive immunization. This assumption was based on evidence suggesting that individuals with higher symptom burden may exhibit stronger cognitive immunization mechanisms, making them less responsive to interventions aimed at promoting expectation adaptation (Berg et al., 2022; Ewen et al., 2022; Kube, Kirchner, et al., 2019). Since cognitive immunization functions as a defense mechanism to maintain dysfunctional expectations, individuals with more severe symptoms may require stronger interventions or repeated exposure to expectation-violating experiences to enhance meaningful change.

## Materials and Method

### Participants

#### Recruitment

Participants were recruited through flyers displayed in public locations such as university buildings, supermarkets, general practitioner practices, pharmacies, and hospitals, as well as through social media groups, the SONA system (Research Participation System of the Philipps-University of Marburg; https://www.sona-systems.com/), and mailing lists at both Philipps-University of Marburg (students and employees) and other universities in German-speaking countries (i.e. Luxemburg and Austria). In addition, support groups for individuals with depression and anxiety disorders were contacted. Recruitment occurred from April 2021 to February 2022. For each participant, one Euro was donated to an organization working to reduce the stigma surrounding mental illness. Psychology students could receive credit points for their bachelor’s degree.

#### Inclusion Criteria

The target sample included individuals with mild depressive and/ or anxiety symptoms. A score between 5-9 on the German version of the Patient Health Questionnaire-9 (PHQ-9; Gräfe et al., 2004) was considered indicative of mild depression. Alternatively, a score between 8-25 on the Beck Anxiety Inventory (BAI; Margraf & Ehlers, 2007), indicating mild to moderate anxiety not clinically relevant, was accepted. These cutoffs were selected for ethical reasons, as the planned behavioral experiments by participants were not controlled by a licensed therapist. Participants were required to be at least 18 years old, have sufficient proficiency in the German language, and have access to a personal email account.

#### Sample Size

The literature suggests that online interventions tend to have small to medium effect sizes (Lintvedt et al., 2013; Sander et al., 2016; Zhou et al., 2016). Prior power analyses were calculated for a MANOVA with repeated measures on within- and between-subject factors, using an effect size of *f* = 0.2 and a power of .80, indicated that 152 participants would be needed. This power analysis was conducted as part of the study’s preregistration (Ewen et al., 2021S) to ensure that the sample size was adequate for detecting meaningful effects given the expected variance in cognitive immunization processes. The selected parameters were based on previous research on online psychological interventions with comparable effect sizes.

#### Study Design

**Figure 1:**
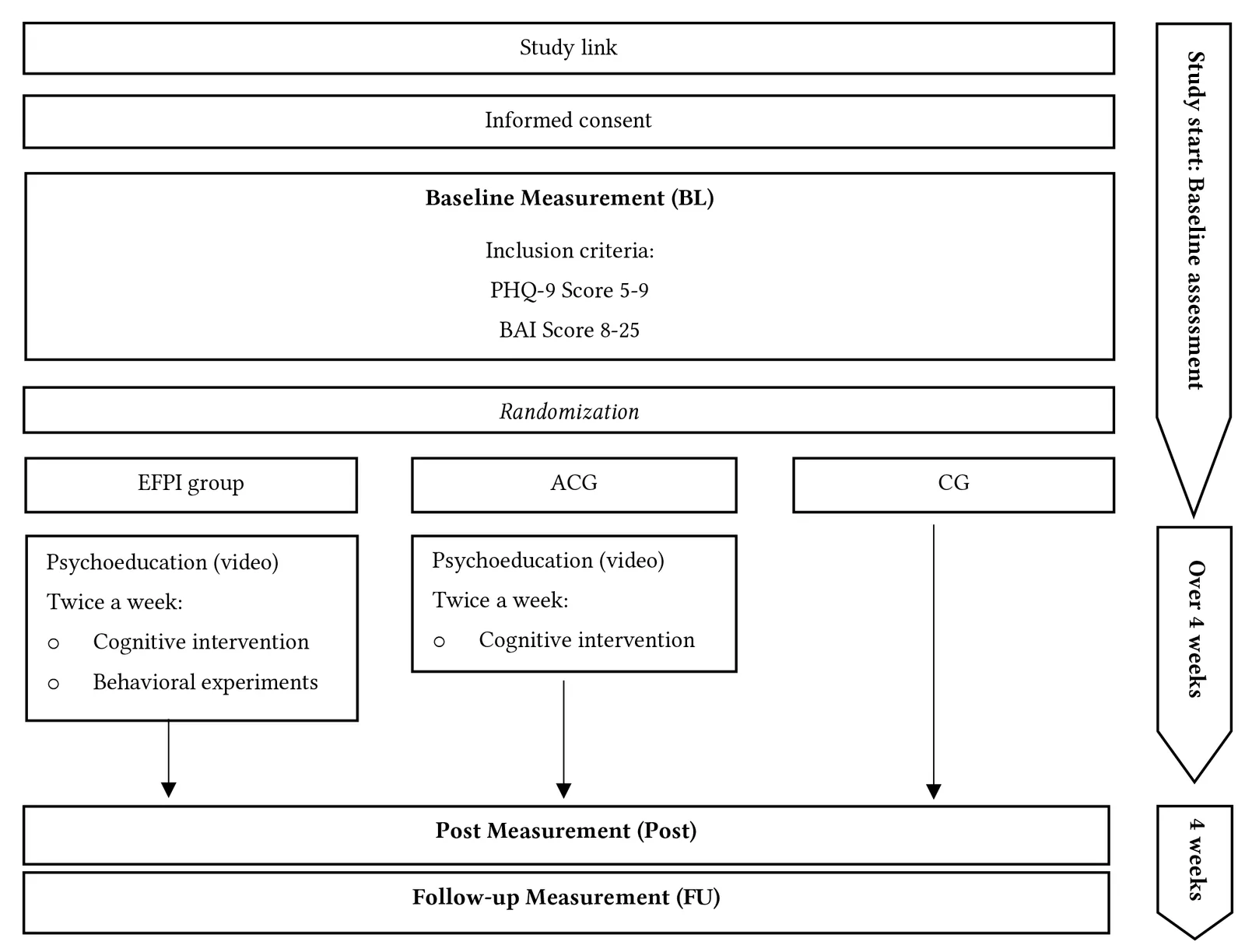
Procedure of the Experiment *Note.* PHQ-9 = Patient Health Questionnaire; BAI = Beck Anxiety Inventory; BL = Baseline measurement; Post = Post measurement; FU = Follow-up measurement; EFPI = expectation focused psychological interventions; ACG = active control group; CG = control group.

The study was conducted entirely online using the survey platform formr (Arslan et al., 2020). At each measurement point, the questionnaires were automatically sent via email to participants through formr. Email addresses were collected at the beginning of the study and stored pseudonymously by the program. Upon clicking on the study link, participants were directed to the study information and informed consent page, which they had to agree to before proceeding. Participants were then automatically randomized by the program (Arslan et al., 2020) into one of three groups: the EFPI group, the ACG, or the CG (see below). Additionally, all the participants were asked to complete baseline questionnaires (see variables for details). After the baseline assessment, the procedures varied among the groups (see Figure 1). For the EFPI group, a psychoeducation video was presented immediately after the completion of the baseline questionnaires. This video covered the role of expectations in shaping behavior and emotions, as well as the impact of dysfunctional expectations, those that contribute to dysfunctional behaviors and long-term negative consequences. Three days later, participants were for the first time introduced to behavioral experiments, designed to test their burdensome situation-specific expectations (see Instructions, Table A1, Appendix). 'Situation-specific expectations' refer to beliefs about particular situations or events that may be unrealistic or rigid, contributing to maladaptive responses to those situations. The chosen expectation had to be very specific and testable over the following three days. Participants documented the planned experiment, their specific expectation regarding the situation, and their level of belief in this expectation (rated from 0-100%). They also recorded the associated emotion. Furthermore, they were asked to reflect on their future behavior in the given situation by considering how their actions could either confirm or disconfirm the expectation (*How can I confirm/disconfirm the expectation through my behavior?*). After three days, participants evaluated the experiment and planned a new one. This process was repeated twice a week for four weeks.

For the ACG, the same psychoeducation video was shown. Participants received a standardized psychoeducational intervention aimed at promoting cognitive restructuring, but without the active expectation testing component. The intervention focused on providing participants with knowledge about cognitive biases and common distortions, aiming to reduce maladaptive thinking patterns. This group was chosen to account for the effects of general psychoeducation, and we expected that any changes in expectations in this group would be less pronounced compared to the EFPI group, given the lack of behavioral experimentation. They were asked every three days, over four weeks, to document their burdensome expectations in the days between the questionnaires and to evaluate them as helpful or unhelpful, as well as noting the associated emotions and behaviors.

The CG did not receive any psychoeducation or interim questionnaires.

After four weeks, all groups completed the post-questionnaires. A follow-up measurement was conducted four weeks later.

### Materials

#### Psychoeducation Video

The animated video using the program Powtoon (Puspitarini et al., 2019) guided participants through the five key questions (Anne-Catherine Ewen, 2021; https://www.youtube.com/watch?v=q_L56tmckos): *What are expectations? Why do humans have expectations? How do expectations arise? Why do certain expectations remain stable? When are expectations causing problems?* The questions were addressed using the basic principles of the ViolEx model and psychoeducational content based on Rief and Glombiewski (2016). The video explained that expectations were future-directed thoughts that could be neutral, positive, or negative and interact with one’s environment. They were arising automatically and were not always consciously recognized. Expectations were shaped by experience, social influences, and individual differences. The evolutionary advantage of planning behavior to prevent harm was highlighted. While the stability of functional expectations would be adaptive, the persistence of dysfunctional expectations could lead to distress by reinforcing avoidance behaviors. Avoidance and immunization processes contribute to the rigidity of expectations, making them resistant to change. Avoidance reduces exposure to corrective experiences, leading to a distorted perception of reality. Even when an expectation is violated, the brain may dismiss the event as insignificant, preventing the correction of distorted perceptions of reality. The video emphasized the importance of consciously observing negative, burdensome, dysfunctional expectations and periodically testing them.

#### Cognitive Intervention

The cognitive intervention was implemented in both the EFPI and ACG groups. It was based on the SORC model, commonly used in cognitive-behavioral therapy (Borg-Laufs, 2020), which facilitates a structured analysis of situations by identifying thoughts, physical sensations, emotions, behaviors, and consequences. Participants selected a burdensome expectation they had in the past three days. They were asked to identify the emotions and the physical sensations triggered by this specific expectation, describe their behavioral response, and reflect on its consequences. The cognitive intervention aimed to help participants become aware of their expectations, understand their influence on emotions and behavior, and encourage a more flexible adaptation of expectations over time. In the EFPI group, this intervention was combined with behavioral experiments to actively test and modify expectations, while in the ACG group, participants engaged only in cognitive reflection without direct behavioral testing. The intervention was designed to reduce cognitive immunization and promote more adaptive expectation processes.

#### Behavioral Experiments

Behavioral experiments were conducted exclusively by the EFPI group. The instructions for the behavioral experiments were adapted from Rief and Glombiewski (2016). Participants selected a specific negative or burdensome expectation and formulated it in a precise, situation-specific manner to ensure testability over the next three days. They rated their belief in the expectation (0-100%) and identified the associated emotion. Additionally, they reflected on their potential behavior in the given situation, considering how they could act in ways that either confirmed or disconfirmed their expectation (*How can I behave to confirm my expectation? How can I behave to disconfirm my expectation?*). A key aim of the behavioral experiments was to counter cognitive immunization by actively engaging participants in testing their expectations. By requiring them to plan and execute experiments that challenge their expectations, the intervention sought to make it more difficult for participants to dismiss disconfirming experiences as mere exceptions. This structured approach was intended to promote a more flexible adaptation of expectations. After three days, they reassessed their belief in the expectation, documented any perceived evidence supporting or contradicting it, and considered alternative interpretations of the situation.

### Measures

#### Demographics

Age, gender, nationality, education, profession, current and past diagnosed mental disorder, and current and past psychotherapeutic treatment were assessed.

#### Cognitive Immunization

The Immunization Scale (IMS; Ewen et al., 2022) was chosen as the primary outcome measure because it assesses key mechanisms involved in expectation persistence, making it a relevant indicator of cognitive immunization. The IMS consists of three subscales: (1) Cognitive Immunization, which measures reappraisal strategies used to maintain expectations despite disconfirming experiences, (2) Negative Expectations, capturing the persistence of negative beliefs that often accompany cognitive immunization, and (3) Assimilation, referring to the integration of expectation-violating information without fundamentally altering the expectation. While the Cognitive Immunization subscale directly measures immunization processes, the other two subscales assess related mechanisms that sustain rigid expectations, aligning with the ViolEx model. The IMS was validated through factor analyses and demonstrated excellent internal consistency (Cronbach’s α = .94). Significant correlations with depression, anxiety, and psychological flexibility further support its construct validity. Cognitive immunization was selected as the primary outcome instead of direct expectation change because expectations are highly specific and individual, making it difficult to systematically assess whether a particular expectation was tested and updated. Instead, we focused on cognitive immunization as the assumed underlying mechanism, as it represents a generalized process by which individuals maintain their expectations despite disconfirming evidence. Depressive and anxiety symptoms were considered moderators rather than primary outcomes because the study aimed to investigate mechanisms of expectation maintenance and change rather than direct symptom reduction.

#### Psychopathology

Depressive symptoms were assessed using the Patient Health Questionnaire – German Version, section PHQ-9 (Gräfe et al., 2004). This 9-item scale primarily evaluates depression criteria as defined by the Diagnostic and Statistical Manual of Mental Disorders DSM-IV. Severity levels are classified as follows: a sum score of 1 to 4 indicates minimal depressive symptoms, 5 to 9 mild, 10 to 14 moderate, and 15 to 27 severe depressive symptoms. Anxiety levels were measured using the Beck Anxiety Inventory BAI (Margraf & Ehlers, 2007), a 21-item scale. A sum score of 0 to 7 indicates minimal anxiety, 8 to 15 mild, 16 to 25 moderate, and 26 to 63 severe anxiety symptoms. A sum score of 26 or higher is considered clinically relevant anxiety.

### Ethics

The study was approved by the local ethics committee of the Department of Psychology at Philipps-University of Marburg (reference number 2020-84k). The study was preregistered on the Open Science Framework (https://osf.io/kvuj7).

### Statistical Analyses

All analyses were conducted using RStudio version 1.2.5042 (Posit PBC, 2009-2020). To account for missing values at the second and third assessment time points, incomplete sum scores of the dependent variables were estimated using multiple imputation (Donders et al., 2006) with the MICE package (van Buuren & Groothuis-Oudshoorn, 2011).

Before conducting the analyses, the Mahalanobis distance was calculated and compared against a χ^2^-cut-off of α = .001, revealing no outliers. Mixed-effects analyses were performed using the *lme4* package (Bates et al., 2018) and *lmerTest* (Kuznetsova et al., 2015). The interaction term time*group was included as a predictor, while participant intercepts and slopes were included as random effects, allowing trends over time to vary for each participant. Analyses of variance ANOVA were calculated for an overview of fixed effects. The package emmeans (Lenth et al., 2018) was used to compute contrasts. The results of the mixed linear models (LLM) were provided by the output of the *tab_model* function of the *sjPlot* package (Lüdecke, 2024). For moderator analyses, moderator variables were included as a triple interaction into the model. The models with and without the moderating term were compared using the Chi-square difference test. Homoscedasticity and normality were checked through residual plots, which exhibited the expected pattern. Due to a high number of missing values, the number of imputations was set to 50 (Bodner, 2008; White et al., 2011). The missing values were assumed to be missing at random (MAR), meaning that their occurrence was independent of the values themselves. The baseline assessment had no missing values (*n* = 1,218), while the second and third assessment time points had 54% and 66% missing values, respectively.

## Results

### Sample Characteristics

**Figure 2:**
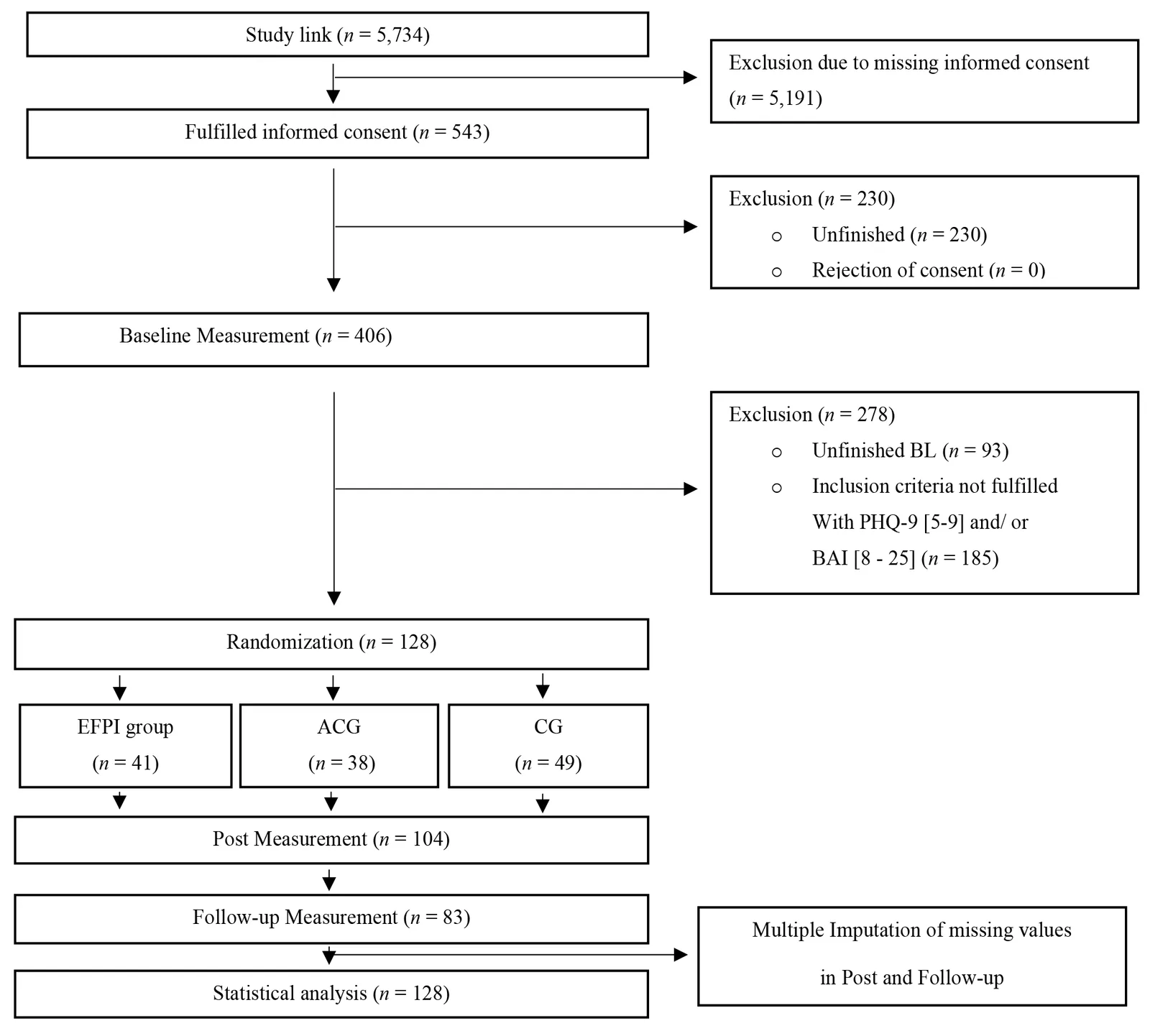
Flow Chart *Note. n* = number of participants; PHQ-9 = Patient Health Questionnaire; BAI = Beck Anxiety Inventory; BL = Baseline measurement; Post = Post measurement; FU = Follow-up measurement; EFPI = expectation focused psychological interventions; ACG = active control group; CG = control group.

A total of 543 individuals agreed to the informed consent. However, 230 participants were excluded due to incomplete baseline assessments, and 185 participants did not meet the inclusion criteria, resulting in a final sample of 128 participants. Among these, 41 participants were allocated to the EFPI group, 38 to the ACG, and 49 to the CG (see Figure 2).

The mean age of the sample was 27 (*SD* = 11.46). Most of the participants were female (82.81%), while 16.41% identified as male and 0.78% as other. Most participants (91.41%) held German nationality. Higher education, defined as holding a university degree, was reported by 24.22% of the sample, while 78.13% were current university students. A history of diagnosed mental disorders was reported by 21.88% of participants, with 8.59% indicating a current diagnosis. Furthermore, 33.59% reported having received psychotherapy at some point in their lives, and 12.50% were engaged in psychotherapeutic treatment during the study period. The means and standard deviations of the assessed questionnaires, comparing imputed and non-imputed data for the post and follow-up measurements, are presented in Table 1.

**Table 1:** Demographics: Mean and Standard Deviations of Different Variables for the Assessment Points Post and Follow-Up

Variable	Total Sample *N* = 128	EFPI *n* = 41	ACG *n* = 38	CG *n* = 49
Age (*M*, *SD*)	28.88 (13.00)	28.07 (12.83)	26.89 (9.89)	31.10 (15.02)
Gender (F, M, N)^a^	106 / 21 / 1	32 / 8 / 1	34 / 4 / 0	40 / 9 / 0
PHQ-9_Baseline_ (*M*, *SD*)	6.27 (1.53)	6.54 (1.34)	6.03 (1.53)	6.24 (1.65)
PHQ-9_Post_ (*M*, *SD*)	6.81 (3.03)	6.71 (2.60)	6.18 (3.20)	6.35 (2.95)
PHQ-9_Follow-up_ (*M*, *SD*)	6.04 (3.04)	5.61 (2.74)	5.89 (3.17)	6.51 (3.16)
BAI_Baseline_ (*M*, *SD*)	10.23 (5.52)	9.37 (5.71)	10.82 (5.28)	10.51 (5.49)
BAI_Post_ (*M*, *SD*)	10.85 (7.89)	11.00 (8.39)	9.29 (6.53)	9.55 (8.40)
BAI_Follow-up_ (*M*, *SD*)	7.89 (7.06)	6.49 (6.49)	7.53 (6.39)	8.98 (7.96)
IMS_Baseline_ (*M*, *SD*)	56.44 (13.45)	55.56 (14.13)	55.53 (12.52)	57.88 (13.73)
IMS_Post_ (*M*, *SD*)	51.31 (13.15)	49.51 (11.84)	50.84 (12.31)	53.18 (14.75)
IMS_Follow-up_ (*M*, *SD*)	48.57 (14.05)	43.88 (10.27)	47.08 (13.40)	53.65 (15.78)

*Note. N* = sample size; *M* = mean; *SD* = standard deviation; PHQ-9 = Patient Health Questionnaire-9; BAI = Beck Anxiety Inventory; IMS = Immunization Scale; EFPI = Expectation Focused Psychological Intervention; ACG = active control group; CG = control group.

^a^F = female, M = male, N = neutral numbers of participants of each group.

### Cognitive Immunization

**Figure 3:**
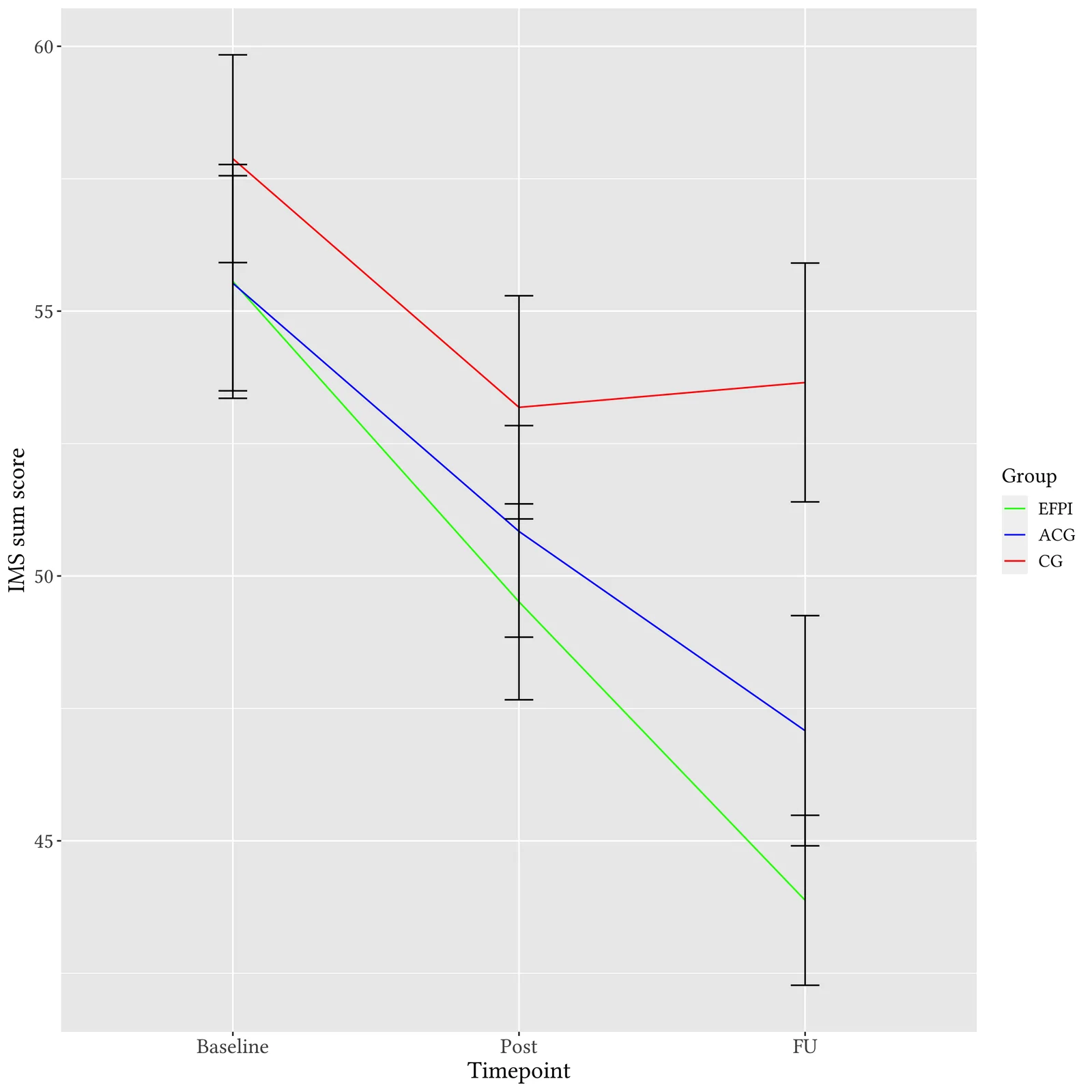
Interaction Plot Mapped for the Immunization Scale *Note.* Figure 3 shows the Immunization Level measured by the IMS over the three measurement time points. The results of the mixed models indicate only one significant interaction at the follow-up timepoint between the groups. IMS = Immunization Scale; Baseline = Baseline measurement; Post = Post measurement; FU = Follow up measurement; EFPI = expectation focused psychological interventions; ACG = active control group; CG = control group.

An ANOVA of the mixed-effects model revealed a significant main effect of timepoint (*F*(2, 122) = 19.96, *p* < .001), whereas the main group (*F*(2,122) = 2.21, *p* = .114) was not significant. The interaction of timepoint and group (*F*(4,122) = 2.12, *p* = .082) was not significant. The interaction plot can be seen in Figure 3.

The results of the linear mixed-effects model further revealed the main significant effect of timepoint, indicating a decrease in the IMS score at the post and follow-up measurement compared to the baseline measurement (post: β = -6.05, 95% CI [-9.90, -2.19], *p* = .002; follow-up: β = - 11.64, 95% CI [-16.00, -7.28], *p* < .001). No significant effect was found for the group variable (ACG: β = -0.18, 95% CI [-6.26, 5.89], *p* = .953; CG: β = 1.97, 95% CI [-3.74, 7.67], *p* = .498), nor were any significant interactions observed between the timepoint and group, while a significant interaction emerged for the follow-up timepoint and the CG (β = 7.43, 95% CI [1.52, 13.35], *p* = .014; see Table 2) indicating that the change in the IMS score over the timepoints differs significantly between the control group and the other groups.

**Table 2:** Output of Mixed Model Including Immunization Scale, Timepoint and Group

Predictors	Estimates	95% CI	*p*
Intercept	55.59	51.38 – 59.80	**< .001**
Post	-6.05	-9.90 – -2.19	**.002**
Follow-up	-11.64	-16.00 – -7.28	**< .001**
ACG	-0.18	-6.26 – 5.89	.953
CG	1.97	-3.74 – 7.67	.498
Post * ACG	1.61	-3.95 – 7.17	.569
Follow-up * ACG	3.65	-2.65 – 9.94	.255
Post * CG	1.38	-3.84 – 6.61	.603
Follow-up * CG	7.43	1.52 – 13.35	**.014**
Random Effects			
σ^2^	52.01		
τ_00_	132.35		
τ_11Post_	51.86		
τ_11Follow-up_	94.79		
ρ01	-0.39		
	-0.43		
ICC	0.71		
*N*	125		
Observations	384		
Marginal *R*^2^/Conditional *R*^2^	0.084/0.735		

*Note.* CI = confidence interval; ACG = active control group; CG = control group; σ^2^ = residual variance; τ_00_ = random intercept variance; τ_11_ = random slope variance; ρ01 = correlation between random intercept and slope; ICC = interclass correlation coefficient; *N* = sample size; significant *p*-values (*p* ≤ .05) are shown in bold.

Random effects analysis showed that the variance in the intercepts across sessions (τ00) was 132.35, and the variance in the slopes (τ11) for the post timepoint and the follow-up timepoint was 51.86 and 94.79, respectively. The intraclass correlation coefficient (ICC) was 0.71, indicating that 71% of the total variability in the outcome was attributable to differences between groups and not within individuals. The model's conditional *R*^2^ was 0.735, suggesting that the model explains a substantial proportion of the variability in the outcome and indicating the importance of the grouping structure, while the marginal *R*^2^ was 0.084, indicating that the fixed effects alone explain a smaller proportion of the variability.

### Psychopathology

#### Depressiveness PHQ-9

**Figure 4:**
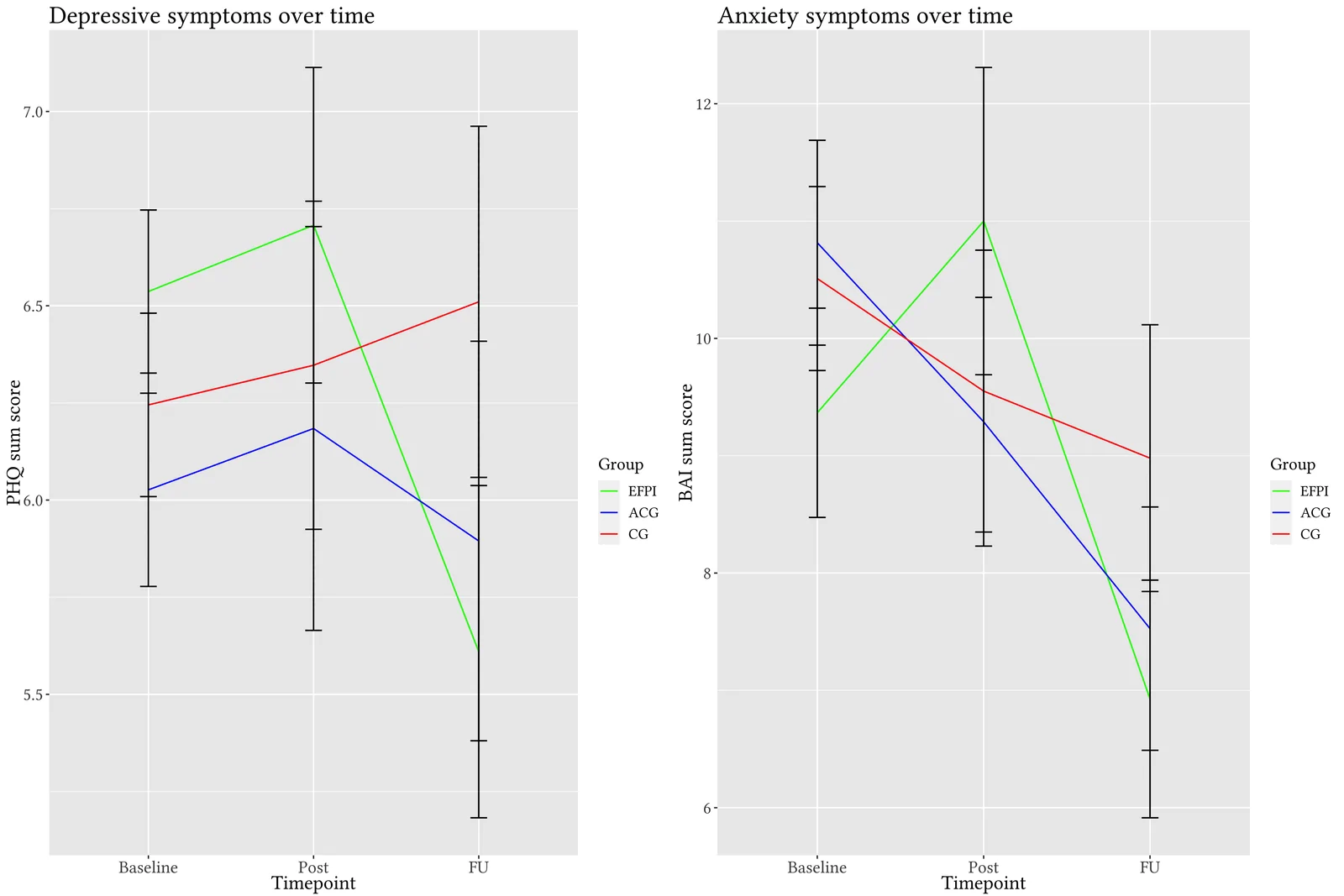
Interaction Plot Mapped for the Depressive and Anxiety Symptoms *Note.* No significant results could be found for the depressive symptoms over the three measurement timepoints (left). A significant interaction could be found for the anxiety symptoms over the three measurement timepoints (right). PHQ-9 = Patient Health Questionnaire; BAI = Beck Anxiety Inventory; Baseline = Baseline measurement; Post = Post measurement; FU = Follow up measurement; EFPI = expectation focused psychological interventions; ACG = active control group; CG = control group.

An ANOVA of the mixed-effects model revealed no significant main (timepoint: *F*(2,121) = 0.18, *p* = .311; group: *F*(2,122) = 0.20, *p* = .820) or interaction (*F*(4,121) = 1.23, *p* = .300) effects (see Figure 4).

The results of the linear mixed-effects model revealed no significant effects of timepoint or group, as well as no significant interactions could be found (see Table 3). The PHQ-9 score did not significantly vary between the groups or measurement timepoints.

**Table 3:** Output of Mixed Model Including Patient Health Questionnaire, Timepoint and Group

Predictors	Estimates	95% CI	*p*
Intercept	6.53	6.06 – 7.00	**< .001**
Post	0.14	-0.71 – 0.99	.744
Follow-up	-0.91	-1.82 – 0.00	.051
ACG	-0.49	-1.17 – 0.19	.159
CG	-0.29	-0.93 – 0.35	.378
Post * ACG	0.08	-1.15 – 1.31	.901
Follow-up * ACG	0.81	-0.51 – 2.12	.231
Post * CG	-0.07	-1.23 – 1.08	.902
Follow-up * CG	1.17	-0.07 – 2.41	.065
Random effects			
σ^2^	1.59		
τ_00_	0.77		
τ_11Post_	4.41		
τ_11Follow-up_	5.54		
ρ01	0.51		
	0.35		
ICC	0.77		
*N*	125		
Observations	384		
Marginal *R*^2^/Conditional *R*^2^	0.014/0.769		

*Note.* CI = confidence interval; ACG = active control group; CG = control group; σ^2^ = residual variance; τ_00_ = random intercept variance; τ_11_ = random slope variance; ρ01 = correlation between random intercept and slope; ICC = interclass correlation coefficient; *N* = sample size; significant *p*-values (*p* ≤ .05) are shown in bold.

Random effects analysis showed that the intercept variance (τ00) was 0.77. The variance in the slopes (τ11) for the post timepoint was 4.41, and for the follow-up timepoint, 5.54. The ICC was 0.77, suggesting that most variability in the PHQ-9 scores is due to between-group differences. The model's conditional *R*^2^ was 0.769, suggesting that the model explains a substantial proportion of the variability in the outcome, while the marginal *R*^2^ was 0.014, indicating that the fixed effects alone explain only a small proportion of the variability.

#### Anxiety BAI

For the calculated ANOVA, a significant main effect for timepoint could be found (*F*(2,125) = 14.46, *p* < .001). The main effect group (*F*(2,122) = 0.20, *p* = .819) was non-significant, the interaction effect (*F*(4,125) = 2.89, *p* = .025) showed a significant result. Contrast analyses looking at the change scores per group, only the EFPI group showed a significant difference between post and follow-up measurement (*t*(119) = 5.03, *p* < .001; see interaction plot Figure 4).

The results of the linear mixed-effects model further revealed the main significant effect of the follow-up timepoint (β = - 2.55, 95% CI [-4.75, -0.36], *p* = .023). No significant effect was found for the group variable (see Table 4), nor were any significant interactions observed between the timepoint and group.

**Table 4:** Output of Mixed Model Including the Beck Anxiety Inventory Timepoint and Group

Predictors	Estimates	95% CI	*p*
Intercept	9.41	7.68 – 11.13	**< .001**
Post	1.51	-0.80 – 3.83	.199
Follow-up	-2.55	-4.75 – -0.36	**.023**
ACG	1.45	-1.03 – 3.94	.251
CG	1.20	-1.14 – 3.53	.314
Post * ACG	-2.98	-6.32 – 0.36	.080
Follow-up * ACG	-0.69	-3.85 – 2.47	.668
Post * CG	-2.40	-5.53 – 0.73	.133
Follow-up * CG	1.07	-1.90 – 4.04	.479
Random effects			
σ^2^	12.67		
τ_00_	18.28		
τ_11Post_	30.49		
τ_11Follow-up_	24.84		
ρ01	0.03		
	-0.14		
ICC	0.74		
*N*	125		
Observations	384		
Marginal *R*^2^/Conditional *R*^2^	0.034/0.744		

*Note.* CI = confidence interval; ACG = active control group; CG = control group; σ^2^ = residual variance; τ_00_ = random intercept variance; τ_11_ = random slope variance; ρ01 = correlation between random intercept and slope; ICC = interclass correlation coefficient; *N* = sample size; significant *p*-values (*p* ≤ .05) are shown in bold.

The random intercept variance (τ00) was 18.28, the random slope variance (τ11) at the post timepoint and the follow-up timepoint was 30.49 and 24.84, respectively. The total variability was attributable to 74% of the differences between the groups. The model's conditional *R*^2^ was 0.744, the marginal *R*^2^ was 0.034.

### Moderations

#### IMS and PHQ-9

The model integrating the depressive symptoms as a moderator (AIC 3013.2) did not explain significantly more variance than the model without a moderator (AIC 3016.5; χ^2^(9) = 16.28, *p* = .061).

In the triple interaction model, ANOVA calculations showed a significant main effect of timepoint (*F*(2,123) = 4.68, *p* = .011) and a significant interaction of timepoint and PHQ-9 (*F*(2,123) = 3.48, *p* = .034). The triple interaction was not significant (*F*(4,123) = 3.48, *p* = .417). The results of the linear mixed-effects model revealed no significant main effects or interactions (see Table 5).

**Table 5:** Output of Mixed Model Including Immunization Scale, Timepoint and Group and Patient Health Questionnaire as Moderator

Predictors	Estimates	95% CI	*p*
Intercept	56.30	35.49 – 77.11	**< .001**
Post	8.61	-10.60 – 27.82	.379
Follow-up	-16.79	-38.78 – 5.21	.134
ACG	-12.80	-40.10 – 14.50	.357
CG	-11.50	-37.13 – 14.13	.378
PHQ-9	-0.11	-3.23 – 3.01	.945
Post * ACG	-3.01	-28.22 – 22.19	.814
Follow-up * ACG	9.44	-19.42 – 38.29	.520
Post * CG	-14.23	-37.89 – 9.43	.238
Follow-up * CG	9.70	-17.39 – 36.79	.482
Post * PHQ-9	-2.24	-5.12 – 0.63	.127
Folloow-up * PHQ-9	0.79	-2.51 – 4.09	.639
ACG * PHQ-9	2.08	-2.14 – 6.31	.332
CG * PHQ-9	2.15	-1.74 – 6.04	.277
Post * ACG * PHQ-9	0.58	-3.32 – 4.48	.770
Follow-up * ACG * PHQ-9	-0.89	-5.35 – 3.57	.695
Post * CG * PHQ-9	2.40	-1.19 – 5.98	.190
Follow-up * CG * PHQ-9	-0.33	-4.44 – 3.78	.876
Random effects			
σ^2^	50.36		
τ_00_	131.26		
τ_11Post_	54.11		
τ_11Follow-up_	102.20		
ρ01	-0.40		
	-0.46		
ICC	0.71		
*N*	125		
Observations	384		
Marginal *R*^2^/Conditional *R*^2^	0.123/0.749		

*Note.* CI = confidence interval; ACG = active control group; CG = control group; PHQ-9 = Patient Health Questionnaire-9; σ^2^ = residual variance; τ_00_ = random intercept variance; τ_11_ = random slope variance; ρ01 = correlation between random intercept and slope; ICC = interclass correlation coefficient; *N* = sample size; significant *p*-values (*p* ≤ .05) are shown in bold.

Random effects analysis showed that the variance in the intercepts across sessions (τ00) was 131.26, and the variance in the slopes (τ11) for the post and the follow-up timepoint was 54.11 and 102.20, respectively. The ICC was 0.71. The model's conditional *R*^2^ was 0.749, and the marginal *R*^2^ was 0.123.

#### IMS and BAI

Integrating anxiety as a moderator in the mixed model (interaction model: AIC 2981.6, triple interaction model: AIC 2974.1) explained the data significantly better (χ^2^(9) = 25.45, *p* = .003). The ANOVA revealed a significant main effect in group (*F*(2,119) = 6.23, *p* = .002). The interaction between timepoint x BAI (*F*(2,122) = 6.43, *p* = .002) and group x BAI (*F*(2,119) = 4.27, *p* = .016) were significant. The triple interaction was non-significant (*F*(4,122) = 0.40, *p* = .806).

The results of the linear mixed effects model further revealed the main significant effect of the CG, indicating an increase in the IMS score in the third group compared to the EFPI group (CG: β = 14.52, 95% CI [3.47, 35.57], *p* = .010). Higher BAI scores were significantly associated with higher IMS scores (β = 1.05, 95% CI [0.35, 1.75], *p* = .003). A significant interaction between the follow-up timepoint and the BAI score was found (β = -0.87, 95% CI [-1.61, -0.13], *p* = .021), indicating that the relationship between the BAI score and the IMS score differs significantly at the follow-up timepoint compared to the other timepoints. Another significant interaction was found between the third group, CG, and the BAI score (β = -1.30, 95% CI [-2.26, -0.33], *p* = .008; see Table 6), indicating that the relationship of the BAI score and the IMS score differs significantly in the CG compared to the other groups.

**Table 6:** Output of Mixed Model Including Score of the Immunization Scale, Timepoint and Group, and Beck Anxiety Inventory as Moderator

Predictors	Estimates	95% CI	*p*
Intercept	45.67	37.99 – 53.35	**< .001**
Post	-0.92	-8.35 – 6.51	.808
Follow-up	-3.43	-11.59 – 4.72	.408
ACG	-3.56	-15.62 – 8.50	.562
CG	14.52	3.47 – 25.57	**.010**
BAI	1.05	0.35 – 1.75	**.003**
Post * ACG	2.99	-8.68 – 14.65	.615
Follow-up * ACG	6.22	-6.58 – 19.03	.340
Post * CG	-3.14	-13.81 – 7.53	.563
Follow-up * CG	4.65	-7.07 – 16.36	.436
Post * BAI	-0.55	-1.22 – 0.13	.114
Follow-up * BAI	-0.87	-1.61 – -0.13	**.021**
ACG * BAI	0.117	-0.86 – 1.21	.742
CG * BAI	-1.30	-2.26 – -0.33	**.008**
Post * ACG * BAI	-0.06	-1.06 – 0.95	.913
Follow-up * ACG * BAI	-0.12	-1.23 – 0.98	.826
Post * CG * BAI	0.49	-0.45 – 1.42	.304
Follow-up * CG * BAI	0.36	-0.67 – 1.38	.492
Random effects			
σ^2^	49.57		
τ_00_	113.50		
τ_11Post_	54.34		
τ_11Follow-up_	85.34		
ρ01	-0.31		
	-0.35		
ICC	0.71		
*N*	125		
Observations	384		
Marginal *R*^2^/Conditional *R*^2^	0.148/0.752		

*Note.* CI = confidence interval; ACG = active control group; CG = control group; PHQ = Patient Health Questionnaire-9; BAI = Beck Anxiety Inventory; σ^2^ = residual variance; τ_00_ = random intercept variance; τ_11_ = random slope variance; ρ01 = correlation between random intercept and slope; ICC = interclass correlation coefficient; *N* = sample size; significant *p*-values (*p* ≤ .05) are shown in bold.

The random intercept variance (τ00) was 113.50, and the random slope variance (τ11) was for the post timepoint and the follow-up timepoint 54.34 and 85.34, respectively. The ICC was 0.71, indicating that 71% of the total variability in the outcome could be explained through the grouping factors and not individual variance. The model's conditional *R*^2^ was 0.752, suggesting that the model explains a substantial proportion of the variability in the outcome, while the marginal *R*^2^ was 0.148.

## Discussion

The primary aim of this study was to evaluate the effectiveness of expectation-focused online interventions in reducing cognitive immunization. The first hypothesis, which proposed a greater reduction in immunization in the EFPI group among the participants with mild symptom severity, can be interpreted as only partially supported. The findings indicate a significant effect of the timepoint on IMS scores with reductions observed at both post-measurement and follow-up. However, no significant main effect of group was found. The results of the mixed models only showed one significant interaction at the follow-up measurement for the CG, suggesting that changes in the IMS scores at the follow-up timepoint differed between the CG and the other groups.

The lack of statistical significance of the interaction between group x timepoint after the ANOVA calculations, as well as the interactions between the ACG and the timepoints in the mixed model results, may be attributed to the smaller sample size than suggested by the a priori power analyses, and the nature of the online intervention, which limits control over participants’ adherence to the interventions. Further studies could address this limitation by incorporating phone consultations with study therapists to enhance adherence. However, the findings are in line with other studies (Kube, Glombiewski, Gall, et al., 2019; Kube, Glombiewski, & Rief, 2019; Rief & Joormann, 2019).

While no significant results were found in the model including depressive symptoms (PHQ-9), the EFPI appeared to have an influence on anxiety symptoms. Looking at the model incorporating anxiety symptoms (BAI), ANOVA calculations revealed a significant main effect of timepoint, and a significant interaction between timepoint and group. Further contrast analyses indicated a significant difference between post-measurement and follow-up within the EFPI group. The results of the mixed models showed as well a significant reduction of the BAI score at the follow-up timepoint. This suggests that EFPI may contribute to a significant reduction in anxiety symptoms. However, no significant interactions between timepoint and group were found. This indicates that after accounting for individual differences (random effects), the interaction effect does not remain strong enough. Further studies with bigger sample sizes should clarify the effects of EFPI on anxiety symptoms.

Moderator analyses further supported the findings that EFPI influences anxiety symptoms. The model, including the BAI, could significantly better explain the data than the model without the BAI as a moderator. Looking at the results of the mixed models, significant moderations could be found at the follow-up timepoint and the third group (CG). Notably, at the follow-up timepoint the relationship between BAI and IMS scores was significantly different, indicating that this relationship varied over time. Additionally, the relationship between BAI and IMS scores was significantly smaller in the CG group compared to other groups. This is in line with previous research on the effectiveness of behavioral experiments in anxiety disorders and the role of exposure in dysfunctional expectations (Craske et al., 2014; McMillan & Lee, 2010).

Overall, differences between the EFPI group and the CG could be found, as well as differences between baseline measurements and follow-up. Moreover, anxiety levels seem to have an influence on the IMS levels and the effect of EFPI on IMS scores. The reduction in immunization at the four-week follow-up suggests that cognitive (ACG) and experience-based (EFPI) interventions may promote enduring changes in information-processing mechanisms. Through a continuous updating process, the discrepancy between preexisting assumptions and reality may decrease - a process that is often impaired in individuals with mental health conditions (Berg et al., 2022). In other words, these interventions may enhance cognitive flexibility by fostering adaptive updating mechanisms, allowing individuals to consider alternative situational interpretations (Fröber et al., 2018; Liknaitzky et al., 2017, 2018; Meiran et al., 2011).

### Strengths and Limitations

This study proposes and tests expectation focused psychological interventions EFPI in a longitudinal online format, offering an economical approach. Until now, cognitive immunization has primarily been examined through complex experimental designs. This study provides initial evidence of the effectiveness of EFPI in reducing immunization over time using a simple self-report questionnaire in a naturalistic setting (Ewen et al., 2022). Additionally, the study contributes to the validation of the IMS, demonstrating that immunization can be influenced following specific interventions. The results suggest that cognitive immunization remains rather stable over time in the CG, highlighting the potential of the interventions. Another advantage of the study design is that it provided implicit support during an uncertain period during the COVID-19 pandemic. Moreover, the findings suggest that EFPI may have a beneficial effect on subclinical or mild anxiety symptoms.

However, the results should be interpreted with caution due to several limitations. A substantial number of participants were lost to post and follow-up assessments, likely due to the lack of therapeutic contact in this online program, which may have facilitated early dropout. Additionally, as participants were contacted via email, the study had limited control over questionnaire completion and participant accessibility. Consequently, data interpretation should be approached with caution, particularly given the need for missing data imputation, even though this method is already well established (Enders, 2017). Another limitation is the restrictive inclusion criteria, which resulted in a relatively small sample size. Additionally, an unequal distribution of female and male participants occurred, with a higher proportion of female participants. This imbalance in gender distribution is common in psychological research, particularly in mental health and online intervention studies, where female individuals tend to be overrepresented (Richmond et al., 2015). While our analyses do not suggest gender-related effects, future studies should aim for a more balanced sample to ensure broader generalizability.

### Future Research and Practical Implications

Future research should include participants with diagnosed mental disorders to evaluate the use of EFPI in psychotherapy over the course of structured Cognitive Behavioral Therapy (Ewen et al., 2023; Wilhelm et al., 2022). Furthermore, future research should explore the potential moderating effects in more depth (i.e., anxiety symptoms). To better understand the underlying mechanisms of cognitive immunization, it would be valuable to investigate cognitive flexibility, as examined in previous studies (Fröber et al., 2018; Liknaitzky et al., 2018). Additionally, future research should explore the connection between expectation management and predictive coding approaches, which is an emerging field of study (Kube et al., 2022). Another important question is whether different mental disorders exhibit similar patterns of increased cognitive immunization and whether EFPI could be effective in these populations as well.

Regarding practical implications, this study could support the integration of EFPI into psychotherapy (Kube, Glombiewski, & Rief, 2019; Rief & Glombiewski, 2016), whereby the influence of EFPI on more severe psychopathology could be analyzed. The interventions appear to have a long-term impact. By actively addressing persistent and dysfunctional expectations in psychotherapy, clinicians may help patients modify rigid cognitive patterns in general. EFPI provides a structured approach to seemingly reduce cognitive immunization, thereby fostering greater flexibility in expectation adaptation.

### Conclusion

This study is the first to evaluate the effectiveness of expectation-focused psychological interventions over a two-month period. Delivered online without offering therapeutic support, these interventions showed an ability to reduce cognitive immunization processes. Since cognitive immunization contributes to the persistence of dysfunctional expectations, reducing these processes may lead to greater adaptability to situational experiences, particularly in individuals with mild depressive and/or anxiety symptoms. Furthermore, the findings suggest a certain relationship between cognitive immunization and anxiety symptoms, with a possible link between the reduction of cognitive immunization and improvement in anxiety symptoms that should be tested in further studies.

## Supplementary Materials

The Supplementary Materials contain the following items:

- *Preregistration* (Ewen et al., 2021S)
- *Appendix:* The appendix includes the instructions of the expectation testing experiments and the table showing the differences between imputed and non-imputed data (Ewen & Wilhelm, 2026S).

EwenA.-C. I.
RiefW.
WilhelmM.
 (2021S). Promote flexibility in expectations (FLEX)
[Preregistration]. PsychOpen. 10.17605/OSF.IO/KVUJ7


EwenA.-C. I.
WilhelmM.
 (2026S). Supplementary materials to "Promoting flexibility in expectations: A randomized-controlled online-intervention study for mild psychopathological symptoms"
[Appendix]. PsychOpen. 10.23668/psycharchives.21808


## Data Availability

Materials and the statistical code supporting this study are available from the corresponding author upon reasonable request. Data sharing may be subject to ethical, legal, or confidentiality restrictions, in line with applicable regulations and participant consent.

## Preregistration

This study has been preregistered on the Open Science Framework OSF (https://osf.io/kvuj7).

## Related Versions

This article is derived from the doctoral thesis of the first author. Some overlap in content is expected, as the thesis served as the basis for the present manuscript. The thesis is publicly accessible (Ewen, 2022). The current article has been revised and adapted for journal publication.

## Reporting Guidelines

This study applied the CONSORT-Statement Guidelines.

## References

[sp1_r1] Ewen A.-C. I. Rief W. Wilhelm M. (2021 S). Promote flexibility in expectations (FLEX) [Preregistration]. PsychOpen. 10.17605/OSF.IO/KVUJ7

[sp1_r2] Ewen A.-C. I. Wilhelm M. (2026 S). Supplementary materials to "Promoting flexibility in expectations: A randomized-controlled online-intervention study for mild psychopathological symptoms" [Appendix]. PsychOpen. 10.23668/psycharchives.21808

